# Acute Large Pericardial Effusion With Haemodynamic Compromise Secondary to Undiagnosed Tuberculosis

**DOI:** 10.7759/cureus.60249

**Published:** 2024-05-14

**Authors:** Zahid Khan, Stephen Hamshere

**Affiliations:** 1 Acute Medicine, Mid and South Essex NHS Foundation Trust, Southend-on-Sea, GBR; 2 Cardiology, Barts Heart Centre, London, GBR; 3 Cardiology and General Medicine, Barking, Havering and Redbridge University Hospitals NHS Trust, London, GBR; 4 Cardiology, Royal Free Hospital, London, GBR

**Keywords:** bronchoscopy, latent tb in the uk, pericardial fluid analysis, emergency pericardiocentesis, rise of latent tb infection, anti-tuberculosis therapy, systemic steroids, pericardial effusion. cardiac tamponade, tuberculous pericardial effusion, : tuberculosis

## Abstract

Tuberculous pericardial effusion is uncommon in the developed countries. However, it remains one of the main causes of presentation with a pericardial presentation with pericardial effusion in the developing world. We present the case of a 24-year-old male patient who presented with a weekly history of diarrhoea, vomiting, shortness of breath and feeling hot. Chest computed tomography revealed a large pericardial effusion with significant haemodynamic compromise. The patient underwent emergency pericardiocentesis, and the pericardial fluid interferon-gamma assay result was positive for tuberculosis. He was unable to tolerate endobronchial biopsy under ultrasound despite heavy sedation and was commenced on anti-tuberculous therapy following a discussion in a multidisciplinary team meeting. He was started on four standard anti-tuberculosis medications, including rifampicin, isoniazid, pyrazinamide, ethambutol and prednisolone. The patient had re-accumulation of pericardial fluid on repeat echocardiography in the first few weeks, which eventually resolved with anti-tuberculous therapy.

## Introduction

Tuberculosis (TB) is a transmissible disease and one of the top 10 leading causes of mortality worldwide [1]. The mortality rate secondary to TB is higher than that of the human immunodeficiency virus (HIV) [1,2]. Tuberculous pericardial effusion mostly develops insidiously, and patients have nonspecific symptoms such as fever, night sweats, fatigue, cough, weight loss, and shortness of breath [3]. Rarely, do these patients present with acute pericardial effusion accumulation with significant haemodynamic compromise. Tuberculous pericarditis is a common cause of constrictive pericarditis and heart failure in developing countries [2]. Several conditions, such as infections, cardiac surgery, chronic pericarditis and mediastinal radiation, can result in pericardial effusion. However, the most common cause in areas with a high prevalence of the disease is TB. A study from South Africa showed that tuberculous pericarditis was the most common reason for referral for diagnostic pericardiocentesis [4]. In contrast, the incidence of tuberculous pericarditis is only 4% in the developed world, but the incidence of this disease is increasing in sub-Saharan Africa due to HIV [4]. The presentation of pericardial TB is variable, and patients can present with fever, weight loss, fatigue, cough, chest pain, shortness of breath and night sweats [5].

## Case presentation

**Figure 1:**
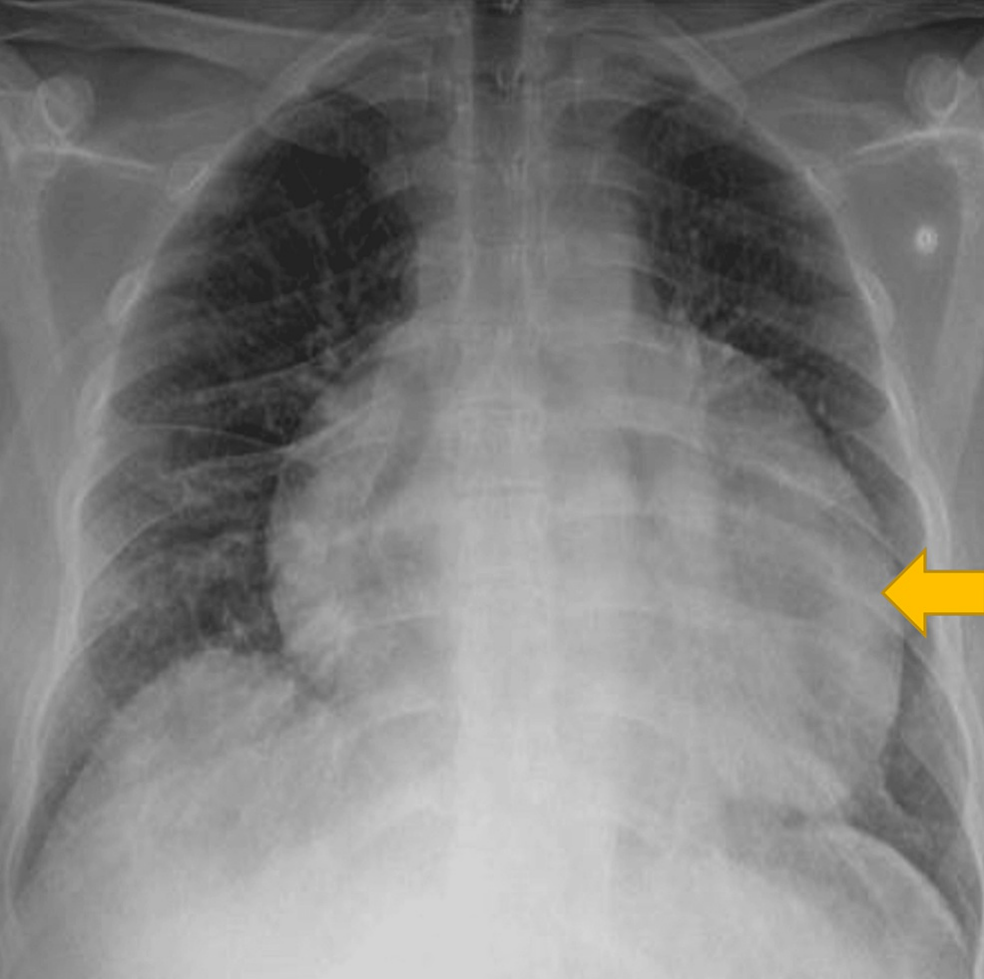
Chest radiography showing a globally increased cardiac shadow in keeping with significant pericardial effusion (yellow arrow).

**Figure 2:**
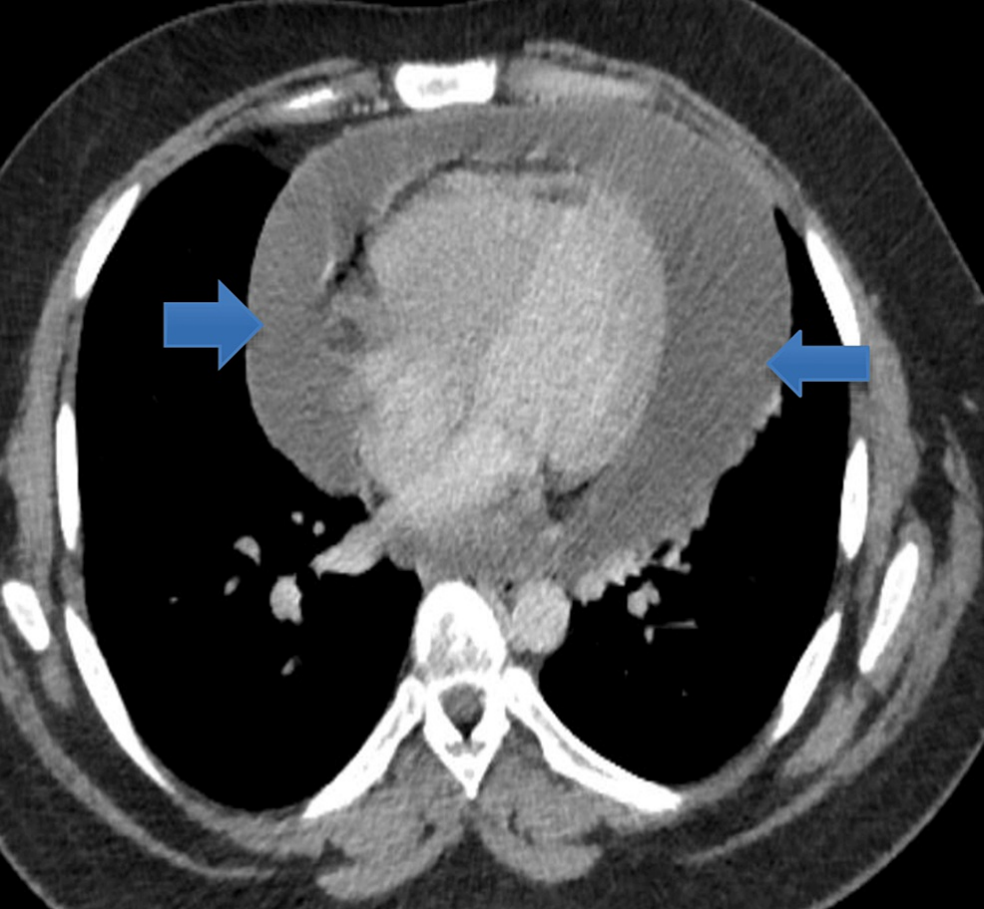
Computed tomography scan of the abdomen and pelvis showing a large pericardial effusion (blue arrows).

A young male patient of Afro-Caribbean origin in his twenties presented to the Accident and Emergency (A&E) Department in London, United Kingdom, with vomiting for eight days and diarrhoea for seven days following eating a takeaway burger eight days back. He started to feel cold and hot after eating the takeaway and had diarrhoea five to six times daily. The patient had reduced oral intake in the past week owing to nausea and vomiting. He developed abdominal pain and shortness of breath three days before hospital admission and consulted his general practitioner. The patient presented to a local hospital with worsening shortness of breath and palpitations. Upon arrival at A&E, he presented with tachycardia and hypotension. Clinical examination revealed tenderness in the right lower quadrant on palpation and tachypnoea. Chest radiography revealed globally increased cardiac shadowing, suggesting significant pericardial effusion (Figure 1). He underwent computed tomography (CT) of the abdomen and pelvis to rule out colitis, which showed mild thickening of the gall bladder and a large pericardial effusion around the left ventricle (LV) measuring approximately 47 mm, with no signs of tamponade (Figure 2). The laboratory results are presented in Table 1.

**Table 1:** Laboratory test results for the patient.

Lab test	Day 1	Day 17	Reference values
Haemoglobin	109	132	120-150 g/L
White cell count	6.0	10.0	4 x 10^9 ^to 10 x 10^9^/L
Platelets	609	467	150x 10^9 ^to 410 x 10^9^/L
Neutrophil	3.2	7.9	2 x 10^9 ^to 7 x 10^9^/L
Urea	6.0	3.9	2.5-7.8 mmol/L
Creatinine	51	49	45-84 umol/L
Sodium	135	140	133-146 mmol/L
Potassium	4.2	4.6	3.5-5.3 mmol/L
C-reactive protein	67	06	0-5 mg/L
Troponin T	14	18	​​​​​0-14 g/L

The vital signs of the patients were as follows: blood pressure (BP), 146/94 mmHg; heart rate (HR), 126 beats per minute (bpm); respiratory rate (RR), 26 breaths per minute; oxygen saturation (SpO_2_), 97% on room air; and temperature (T), 39.4 °C. The patient developed a new oxygen requirement late in the afternoon, and an urgent echocardiogram demonstrated a large pericardial effusion of 3.7 cm around the LV, right atrial collapse in systole and right ventricle collapse in diastole and dilated inferior vena cava size (>2.1 cm), with <50% inspiratory collapse (Videos 1-2).

**Video 1:** Echocardiography parasternal long-axis views showing a large pericardial effusion.

**Video 2:** Echocardiography subcostal view showing large global pericardial effusion.

Sepsis screening, including blood culture and chest radiography, was performed. The patient was transferred to our hospital because of echocardiographic findings and new oxygen requirements. He underwent emergency pericardiocentesis, 770 mL of blood-stained pericardial fluid was aspirated and the pericardial drain was sutured with free drainage for 24 hours. The patient was commenced on intravenous amoxicillin and clavulanic acid 1.2 g three times daily (TDS) and received a stat dose of amikacin based on the microbiologist’s advice. Pericardial fluid was sent for cytology, biochemistry and microbiological testing, including acid-fast bacilli. Repeat echocardiography after pericardiocentesis showed a small localised pericardial effusion approximately 1.25 cm behind the right atrium, 0.54 cm around the left ventricular apex and normal left ventricular function with no signs of tamponade. CT thorax showed a small decrease in the size of the loculated pericardial effusion with increased peripheral enhancement of the pericardial effusion, multiple enlarged mediastinal, hilar and infra-clavicular lymph nodes, and consolidation in the right lower lobe. The patient’s temperature continued to spike despite being on intravenous amoxicillin and clavulanic acid 1.2 g TDS.

The patient was reviewed by both the infectious disease and rheumatology teams and had several negative blood culture results. His autoimmune screen was negative, except for immunoglobulin G (IgG) antibodies for cytomegalovirus (CMV) and Epstein-Barr virus (EBV), suggestive of past infection. Mycoplasma, Legionella, pneumococcal antigen, Coxiella and Brucella tests were negative. The patient did not tolerate endobronchial ultrasound examination despite maximum sedation. Antibiotics were switched to piperacillin with tazobactam 4.5 mg TDS due to continued temperature spikes and lack of clinical improvement from an unclear source of infection. The interferon-gamma assay for TB was positive, suggesting previous exposure to TB. The patient was discussed in a respiratory multidisciplinary meeting and commenced on rifater tablets (isoniazid 300 mg, pyrazinamide 1,800 mg and rifampicin 600 mg) once daily (OD), rifampicin 300 mg OD, pyridoxine 25 mg and ethambutol 1.2 g, and prednisolone 60 mg OD weaning dose to be reduced by 10 mg every week. Repeat echocardiogram a week after commencement of anti-TB therapy showed trivial pericardial effusion 0.23 cm behind the right atrium and 0.2 cm inferolateral to the LV, which increased in size on further echocardiography three weeks later to <0.5 cm near the LV without any right ventricle diastolic collapse and normal left ventricular function. The patient was reviewed by an ophthalmologist for blurred vision before starting ethambutol and was found to have a normal examination.

Differential diagnosis

Possible differential diagnoses include infectious gastroenteritis, acute surgical abdomen, chest infection, TB and malignancy. The patient’s stool samples were negative for infection and the CT scan did not show any evidence of an acute surgical abdominal emergency. The patient underwent chest radiography, which did not show any consolidation. The CT abdomen and pelvic CT did not show any evidence of malignancy, and pericardial fluid analysis was negative for malignant cells, despite the presence of blood-stained pericardial fluid. The pericardial effusion sample was positive for acid-fast bacilli, consistent with tuberculous infection, and he responded well to anti-TB therapy. Finally, blunt chest trauma or surgery can result in significant pericardial effusion, which was not observed in our patient.

Outcome and follow-up

He remains on current anti-TB therapy, except for the weaning of his steroids (5 mg weekly) until completely stopped. He tolerated the therapy well, and his latest echocardiogram showed complete resolution of pericardial effusion. He remains under respiratory clinic follow-up and has been undergoing regular chest radiography and medication reviews.

## Discussion

TB can be both pulmonary and extra-pulmonary, and the latter can often be missed owing to its atypical features. Extra-pulmonary TB most commonly affects the pleura and lymph nodes, followed by the joints and bones, intestines, peritoneum, genitourinary system, and meninges [1,4]. Skeletal TB is the most common form of TB in China, whereas pericardial TB can be caused by retrograde dissemination of Mycobacterium TB from the trachea, bronchi, and lymphatics, or haematogenous spread of primary TB infection [6]. TB should be suspected in patients presenting with pericarditis or pericardial effusion, without a clear cause. The incidence of tuberculous pericardial effusion varies; one study reported that TB accounted for less than 4% of pericardial effusion cases in developed countries, whereas it accounted for 70% of all pericardial effusion cases in a South African study [7]. The clinical presentation of TB pericarditis and pericardial effusion can vary from symptoms, including fever, weight loss, fatigue and night sweats, to completely asymptomatic patients. Eight patients with pericardial effusion developed pericarditis, which is usually divided into four stages: dry, effusive, absorptive and constrictive, caused by scarring [8]. 

The direct Ziehl-Neelsen stain has a poor detection rate of 0% to 42% for acid-fast bacilli, whereas pericardial fluid culture is positive in approximately 55% of cases. Another study reported a higher positive yield of AFB detection for the pericardial fluid culture of approximately 93% compared with 81% and 87% for polymerase chain reaction (PCR) and histology, respectively [9,10]. Pericardiocentesis is recommended for all patients with suspected TB, and a study in South Africa reported the presence of cardiac tamponade in 10% of patients with TB pericardial effusion [11]. Chest radiography may demonstrate features of active pulmonary TB in only 30% of cases and pleural effusion in approximately 40% to 60% of cases [11]. Electrocardiograms (ECGs) may show non-specific T waves, ST segment changes, ST elevation and PR segment deviation in approximately 9% to 11% of cases [11,12]. Other ECG features include micro-voltages, such as complexes <5 mm in limb leads and <10 mm in precordial leads, suggesting large pericardial effusion and tamponade. The presence of electrical alternans is a hallmark of cardiac tamponade on electrocardiography in patients with a large pericardial effusion [11,12]. Echocardiography may reveal pericardial effusion with fibrinous strands in the visceral pericardium, and pericardial thickening can be observed in patients with TB. The pericardial fluid is blood-stained in >80% of patients with TB pericardial effusion, and pericardiocentesis is an absolute indication in patients with tamponade [11,12].

TB pericardial effusion tends to be exudative and is characterised by high protein content and increased leukocyte counts, mainly lymphocytes and monocytes [11,12]. The pericardial effusion in our patient was also haemorrhagic, and the patient had features of cardiac tamponade requiring emergency pericardiocentesis. Few published case reports have described pericardial effusion and pericarditis as the initial presentations of patients with TB [13,14]. The pericardial fluid cultures tested positive for acid-fast bacilli, although the sputum was negative. Our patient had positive pericardial fluid cultures for acid-fast bacilli and did not tolerate bronchoscopy. These patients were treated with a combination of isoniazid (INH) 300 mg, rifampicin (RIF) 600 mg, ethambutol (EMB) 1,500 mg and pyrazinamide (PZA) 2,000 mg. Our patient received the aforementioned treatment along with a weaning dose of steroids and did not show any recurrence of pericardial effusion following successful treatment of TB.

## Conclusions

Tuberculous pericardial effusion is an uncommon initial presentation of TB in the developed world. Physicians should consider TB as a potential diagnosis in patients presenting with pericardial effusion. Echocardiography is useful for identifying patients with cardiac tamponade secondary to massive pericardial effusion. Anti-TB therapy is recommended for patients with tuberculous pericardial effusion. Pericardial effusion recurrence is rare in patients with properly treated TB.
